# Novel Symptom Subgroups in Patients With Irritable Bowel Syndrome Are Associated With Healthcare Utilisation in Secondary and Tertiary Care

**DOI:** 10.1111/apt.70583

**Published:** 2026-02-19

**Authors:** Vivek C. Goodoory, Mais Khasawneh, Christy Riggott, Dipesh H. Vasant, Maria Eugenicos, Imran Aziz, Maura Corsetti, Peter A. Paine, Ryan Danvers, Karen McKinnie, Debbie Bush, Christopher J. Black, Alexander C. Ford

**Affiliations:** ^1^ Leeds Institute of Medical Research at St. James's University of Leeds Leeds UK; ^2^ Leeds Gastroenterology Institute St. James's University Hospital Leeds UK; ^3^ Neurogastroenterology Unit Wythenshawe Hospital, Gastrointestinal Medicine and Surgery, Manchester University NHS Foundation Trust Manchester UK; ^4^ Division of Diabetes, Endocrinology and Gastroenterology University of Manchester Manchester UK; ^5^ Department of Gastroenterology Western General Hospital Edinburgh UK; ^6^ Division of Clinical Medicine School of Medicine and Population Health, University of Sheffield Sheffield UK; ^7^ Department of Gastroenterology Sheffield Teaching Hospitals Sheffield UK; ^8^ Department of Gastroenterology Queen's Medical Centre Nottingham UK; ^9^ Department of Gastroenterology Salford Royal NHS Foundation Trust Manchester UK

**Keywords:** healthcare utilisation, irritable bowel syndrome, latent class analysis, prognosis, subgrouping

## Abstract

**Background & Aims:**

Current classification systems for irritable bowel syndrome (IBS) based on bowel habit do not consider psychological impact. We applied a previously validated latent class analysis (LCA) model to a cohort of patients with IBS in secondary and tertiary care to assess whether it predicted levels of healthcare utilisation.

**Methods:**

We applied our LCA model to a referral population with IBS. As described previously, we assigned cluster membership based on gastrointestinal symptom severity and psychological burden. We assessed demographics, symptom severity and quality of life at baseline and levels of healthcare utilisation during 12 months of longitudinal follow‐up according to cluster.

**Results:**

We recruited 379 patients, of whom 249 (65.7%) met the Rome IV criteria. Those in the four clusters with higher psychological burden had more severe symptoms on the IBS‐SSS and lower quality of life scores (*p* < 0.001 for both). Rates of discharge were generally lower in clusters with higher psychological burden (*p* = 0.05). Rates of prescribing a drug for IBS (*p* = 0.001), the mean number of drugs prescribed for IBS (*p* < 0.001) and the mean number of different drug types prescribed for IBS (*p* < 0.001 for trend) were highest in the four clusters with higher psychological burden.

**Conclusions:**

In patients with IBS in secondary and tertiary care, the LCA model identified groups of individuals with more severe symptoms and greater impairments in quality of life at baseline and significantly higher rates of healthcare utilisation during longitudinal follow‐up.

## Graphical abstract

In patients with IBS in secondary and tertiary care, latent class analysis model identified groups of individuals with more severe symptoms and greater impairments in quality of life at baseline and significantly higher rates of healthcare utilisation during longitudinal follow‐up.

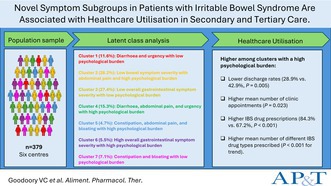

**Abbreviations**
HADS: hospital anxiety and depression scale
IBS: irritable bowel syndrome
IBS‐C: irritable bowel syndrome with constipation
IBS‐D: irritable bowel syndrome with diarrhoea
IBS‐M: irritable bowel syndrome with mixed bowel habits
IBS‐QOL: irritable bowel syndrome quality of life
IBS‐SSS: irritable bowel syndrome severity scoring system
IBS‐U: irritable bowel syndrome unclassified
PHQ‐12: patient health questionnaire‐12
RCT: randomised controlled trial
SD: standard deviation

## 1 Introduction

Irritable bowel syndrome (IBS) is a highly prevalent disorder [1, 2], characterised by abdominal pain in association with either abnormal stool form or frequency [3]. IBS has an impact on healthcare systems, due to management costs [4], society, due to its impact on work [5] and the individual, due to its effect on quality of life and other aspects of social functioning [5, 6].

The pathophysiology of IBS is thought to reflect abnormal gut‐brain communication [7]. Hence, the condition is viewed as a disorder of gut‐brain interaction and this nomenclature has replaced the term ‘functional’, which otherwise suggests there is no underlying cause for symptoms [8]. This is compatible with the observation that psychological health can play an important role in the development and persistence of symptoms [9, 10, 11, 12, 13]. In acknowledgment of this, the Rome Foundation developed the multi‐dimensional clinical profile. This is a framework that includes the assessment of psychological factors and impact of the illness, as well as clinical symptoms, building a unique clinical profile for each patient, with the aim being to facilitate more personalised management [14].

This approach is supported by multiple studies from independent groups of investigators that have used a statistical technique called latent class analysis (LCA) to subgroup people with IBS using combinations of gastrointestinal and psychological symptoms [15, 16, 17, 18, 19, 20]. The results of these studies are similar. There are clusters of individuals with IBS in whom gastrointestinal symptoms predominate, clusters in whom gastrointestinal symptoms are milder and psychological symptoms predominate, and clusters in whom gastrointestinal symptoms are more severe and are part of a broader picture, including symptoms of anxiety, depression or somatisation. During longitudinal follow‐up of one LCA study, those in clusters with higher levels of psychological symptom reporting had more severe IBS throughout, received a higher number of subsequent treatments for their IBS, and were more likely to consult a doctor with their IBS than people in clusters with lower levels of psychological symptom‐reporting [21].

Although one of these studies was conducted among a referral population [17], all the others have recruited people with IBS from the community with a self‐reported diagnosis of the condition, or individuals in the general population who meet current symptom‐based criteria for IBS [15, 16, 19]. Whether these clusters are observed in patients with IBS in referral populations, and whether they predict higher levels of healthcare utilisation, is unknown. We, therefore, applied our previous LCA model [15], this time to a cohort of patients with IBS in secondary and tertiary care. We hypothesised that those in clusters with higher levels of psychological symptoms would be less likely to be discharged from follow‐up, would be more likely to be investigated, and would cycle through more treatments than those in clusters with lower levels of psychological symptoms.

## 2 Methods

### 2.1 Participants and Setting

The study was conducted in six teaching hospitals in the UK National Health Service, serving diverse populations. Consecutive adult patients (≥ 18 years) referred for the first time from primary care with suspected IBS to the gastroenterology outpatient clinics of these centres were recruited between September 2021 and June 2024. All clinics were run by gastroenterologists with an interest in managing IBS. Patients were classified as having IBS according to diagnosis by the consulting gastroenterologist, where typical symptoms of IBS were reported and there was no organic explanation for these symptoms after investigation according to the diagnostic algorithm recommended by current UK guidelines [22]. There were no exclusion criteria other than an inability to understand written English. Eligible individuals were recruited in‐person, at their index clinic consultation, and completed a questionnaire at enrolment. All responses were stored in an online database. This study was approved by the Health Research Authority and Health and Care Research Wales ethics committee (REC ref.: 21/SC/0147).

### 2.2 Data Collection and Synthesis

#### 2.2.1 Demographic and IBS Symptom Data

Demographic data, including age, sex, lifestyle factors (tobacco and alcohol consumption), ethnicity, marital status and educational level, were collected at baseline. All participants were asked whether their IBS was triggered after an acute enteric infection, as well as to identify their most troublesome symptom from a list of five possibilities, comprising abdominal pain, constipation, diarrhoea, bloating or urgency.

We also applied a more stringent definition of IBS, where the presence of IBS was defined using the Rome IV criteria [3], assigning presence or absence of Rome IV‐defined IBS among all individuals according to the Rome IV questionnaire [23], and with no organic explanation for these symptoms after investigation according to the current UK diagnostic algorithm [22]. IBS was subtyped according to the predominant stool form on days when stools were abnormal using the Bristol stool form scale, as recommended [23].

#### 2.2.2 IBS Symptom Severity, Mood, Somatic Symptoms and IBS‐Specific Quality of Life

IBS symptom severity was assessed using the IBS severity scoring system (IBS‐SSS) [24], which is scored from 0 to 500 points; < 75 points indicates remission, 75–174 points mild, 175–299 points moderate and 300–500 points severe symptoms.

Symptoms of anxiety or depression were assessed using the hospital anxiety and depression scale (HADS) [25], with a total score of 0 to 21 for either anxiety or depression. Symptom severity for each was categorised into normal (total HADS depression or anxiety score 0–7), borderline normal (8–10) or abnormal (≥ 11), as recommended [25].

Somatic symptom‐reporting data were collected using the patient health questionnaire‐12 (PHQ‐12) [26], derived from the validated PHQ‐15 [27]. The total PHQ‐12 score ranges from 0 to 24. Severity was categorised into high (total PHQ‐12 ≥ 13), medium (8–12), low (4–7) or minimal (≤ 3).

The irritable bowel syndrome quality of life (IBS‐QOL), a validated IBS‐specific questionnaire, was used to measure health‐related quality of life [28, 29]. It consists of 34 items ranked on a 5‐point Likert scale, ranging from 0 to 4. The total possible score is 0–136, with lower scores indicating better quality of life. Scores were transformed to a 0 to 100‐point scale with zero indicating worst quality of life and 100 indicating best quality of life [28].

#### 2.2.3 Assigning Patients to the Seven‐Cluster Model

We applied the LCA model that we derived and validated previously to all individuals meeting criteria for IBS [15]. LCA is a method of structural equation modelling used to identify unobserved groups or latent classes, within observed multivariate data [30]. As the syntax for our LCA model derived previously is stored, it can be applied easily to other datasets that collect the same variables. Details of the variables used in the model are provided in Table S1.

In our original study this model generated seven clusters [15], as detailed in Figure S1. These were as follows: diarrhoea and urgency with low psychological burden (cluster 1, Figure S1A), diarrhoea, abdominal pain and urgency with high psychological burden (cluster 4, Figure S1D), constipation and bloating with low psychological burden (cluster 7, Figure S1G), constipation, abdominal pain and bloating with high psychological burden (cluster 5, Figure S1E), low overall gastrointestinal symptom severity with low psychological burden (cluster 3, Figure S1C), low bowel symptom severity with abdominal pain and high psychological burden (cluster 2, Figure S1B), and high overall gastrointestinal symptom severity with high psychological burden (cluster 6, Figure S1F).

#### 2.2.4 Healthcare Use Among Individuals With IBS According to Cluster

We collected data on healthcare usage related to each patient's IBS over the 12 months following recruitment, via review of their electronic medical records in each participating centre. We recorded the number of appointments with a gastroenterologist for IBS, referrals to dietitians, gut‐directed hypnotherapists or psychologists, number of investigations (endoscopies, radiological investigations, including 23‐seleno‐25‐homo‐tauro‐cholic acid scans or lower gastrointestinal physiological testing) and the number and type of prescribed drugs over the 12 months.

### 2.3 Statistical Analysis

We compared categorical variables, such as sex, lifestyle factors, ethnicity, marital status, educational level, IBS subtype, most bothersome symptom and IBS symptom severity, between each of the seven clusters using a *χ* ^2^ test. We compared differences in continuous variables, such as age and IBS‐QOL scores, using one‐way analysis of variance (ANOVA). To assess healthcare use associated with each of the seven clusters, we compared rates of discharge from clinic and rates of referral to dietitians, gut‐directed hypnotherapists or psychologists between clusters during the 12 months of follow‐up using a *χ* ^2^ test. We compared the number of appointments for IBS, the number of investigations and the number of different drugs prescribed between clusters during the 12 months of follow‐up using a one‐way ANOVA. We repeated these analyses according to whether individuals were in a cluster with a high psychological burden (clusters 2, 4, 5 and 6) or a low psychological burden (clusters 1, 3 and 7) using a χ^2^ test for categorical variables and an independent samples *t*‐test for continuous data.

Due to multiple univariate comparisons according to cluster, we considered a 2‐tailed *p* value of < 0.01 as indicating statistical significance. For analyses related to healthcare use in IBS according to cluster, as this was the main hypothesis of the study, we considered a 2‐tailed *p* value of < 0.05 as indicating statistical significance. All analyses were performed using SPSS for Windows (version 30.0 SPSS Inc., Chicago, IL, USA).

## 3 Results

In total, we recruited 379 patients (mean age 42.3 years (range 18–83 years), 314 (82.8%) female, 339 (89.7%) White) with IBS diagnosed by a gastroenterologist and confirmed after investigation according to the diagnostic algorithm recommended by national guidelines. Of these, 249 (65.7%) (mean age 40.4 years (range 18–78 years), 201 (80.7%) female, 221 (89.1%) White) also met Rome IV criteria. Differences in characteristics between these two populations are provided in Table 1 but, as these were overlapping, no formal statistical testing was undertaken. Differences in demographics and IBS symptom data between those diagnosed with IBS by a gastroenterologist and those meeting Rome IV criteria were minimal, with the exception that fewer patients with Rome IV IBS were in remission on the IBS‐SSS and mean IBS‐SSS scores were generally higher in those meeting Rome IV criteria. Among those with Rome IV IBS, higher numbers had abnormal HADS‐anxiety scores and had high levels of somatisation on the PHQ‐12, and mean IBS‐QOL scores were lower. Finally, more individuals meeting Rome IV criteria were in one of the clusters with a high psychological burden (clusters 2, 4, 5 and 6). As characteristics of those with IBS diagnosed by a gastroenterologist were generally similar to those diagnosed by a gastroenterologist who also met Rome IV criteria, we included data from all 379 recruited patients in all subsequent analyses.

**TABLE 1:** Baseline characteristics of individuals with IBS diagnosed by a gastroenterologist and confirmed after investigation according to the diagnostic algorithm recommended by National guidelines, compared with those who also met the Rome IV criteria.

	IBS diagnosed by a gastroenterologist (*n* = 379)	IBS diagnosed by a gastroenterologist and met Rome IV criteria (*n* = 249)
Mean age (SD)	42.3 (15.1)	40.4 (14.5)
Female (%)	314 (82.8)	201 (80.7)
Smoker (%)	49 (12.9)	38 (15.3)
Alcohol use (%)	214 (56.5)	136 (54.6)
White ethnicity (%)	339 (89.7)	221 (89.1)
Married (%)	183 (48.3)	121 (48.6)
University or postgraduate level of education (%)	150 (39.6)	94 (37.8)
IBS subtype (%)		
IBS‐C	92 (24.3)	55 (22.1)
IBS‐D	120 (31.7)	83 (33.3)
IBS‐M	158 (41.7)	109 (43.8)
IBS‐U	9 (2.4)	2 (0.8)
IBS after acute enteric infection (%)	34 (9.0)	27 (10.8)
Most troublesome symptom (%)		
Abdominal pain	140 (36.9)	100 (40.2)
Constipation	41 (10.8)	21 (8.4)
Diarrhoea	52 (13.7)	34 (13.7)
Bloating/distension	89 (23.5)	56 (22.5)
Urgency	57 (15.0)	38 (15.3)
IBS‐SSS severity (%)		
Remission	13 (3.4)	2 (0.8)
Mild	46 (12.1)	21 (8.4)
Moderate	194 (51.2)	132 (53.0)
Severe	126 (33.2)	94 (37.8)
Mean IBS‐SSS score (SD)	258.0 (85.1)	275.9 (75.4)
Met Rome IV criteria for IBS (%)	249 (65.7)	249 (100)
HADS‐anxiety categories (%)		
Normal	126 (33.2)	75 (30.1)
Borderline abnormal	91 (24.0)	57 (22.9)
Abnormal	162 (42.7)	117 (47.0)
Mean HADS‐anxiety score (SD)	9.8 (4.9)	10.3 (5.0)
HADS‐depression categories (%)		
Normal	225 (59.5)	144 (57.8)
Borderline abnormal	80 (21.2)	53 (21.3)
Abnormal	73 (19.3)	52 (20.9)
Mean HADS‐depression score (SD)	6.9 (4.6)	7.3 (4.6)
PHQ‐12 severity (%)		
Low	22 (5.8)	10 (4.0)
Mild	98 (26.0)	53 (21.5)
Moderate	167 (44.3)	114 (46.2)
Severe	90 (23.9)	70 (28.3)
Mean PHQ‐12 score (SD)	9.8 (4.3)	10.4 (4.1)
Mean IBS‐QOL score (SD)	51.8 (24.0)	47.0 (23.2)
Cluster		
1: Diarrhoea and urgency with low psychological burden	44 (11.6)	34 (13.7)
2: Low bowel symptom severity with abdominal pain and high psychological burden	107 (28.2)	67 (26.9)
3: Low overall gastrointestinal symptom severity with low psychological burden	104 (27.4)	42 (16.9)
4: Diarrhoea, abdominal pain and urgency with high psychological burden	58 (15.3)	51 (20.5)
5: Constipation, abdominal pain and bloating with high psychological burden	18 (4.7)	18 (7.2)
6: High overall gastrointestinal symptom severity with high psychological burden	21 (5.5)	19 (7.6)
7: Constipation and bloating with low psychological burden	27 (7.1)	18 (7.2)

### 3.1 Baseline Characteristics According to Cluster

In terms of demographics, those in clusters with a low psychological burden (clusters 1, 3 and 7) were more likely to drink alcohol (*p* < 0.001 for trend) (Table 2), but there were no other significant differences. As would be expected, IBS subtype, most troublesome symptom and IBS symptom severity varied significantly according to cluster (*p* < 0.001 for trend for all). Most patients in clusters 1 and 4 met criteria for IBS‐D or IBS‐M; most in clusters 5 and 7 met criteria for IBS‐C; and most in clusters 2, 3 and 6 met criteria for IBS‐M. Diarrhoea and abdominal pain were reported as the most troublesome symptoms in cluster 1, abdominal pain in cluster 2, abdominal pain and urgency in cluster 4, abdominal pain, constipation and bloating/distension in cluster 5 and bloating/distension and constipation in cluster 7. Clusters 3 and 6, however, were more heterogeneous in terms of most troublesome symptom. More patients in clusters with a high psychological burden (clusters 2, 4, 5 and 6) had severe symptoms on the IBS‐SSS; mean IBS‐SSS scores were highest in these clusters (*p* < 0.001 for trend for both). Similarly, HADS‐anxiety and HADS‐depression scores were more likely to be abnormal among patients in these four clusters; more of these patients had high levels of somatisation; and mean HADS‐anxiety, HADS‐depression and PHQ‐12 scores were all highest in these clusters (*p* < 0.001 for trend for all). Lastly, IBS‐QOL scores were all lower in clusters 2, 4, 5 and 6 (*p* < 0.001 for trend).

**TABLE 2:** Baseline characteristics of individuals with IBS according to cluster.

	Cluster 1: diarrhoea and urgency with low psychological burden (*n* = 44)	Cluster 2: Low bowel symptom severity with abdominal pain and high psychological burden (*n* = 107)	Cluster 3: Low overall gastrointestinal symptom severity with low psychological burden (*n* = 104)	Cluster 4: diarrhoea, abdominal pain and urgency with high psychological burden (*n* = 58)	Cluster 5: constipation, abdominal pain and bloating with high psychological burden (*n* = 18)	Cluster 6: High overall gastrointestinal symptom severity with high psychological burden (*n* = 21)	Cluster 7: constipation and bloating with low psychological burden (*n* = 27)	*p* *
Mean age (SD)	43.5 (12.4)	39.6 (14.7)	44.4 (17.1)	41.3 (14.3)	43.9 (10.1)	44.4 (15.8)	42.0 (15.8)	0.37
Female (%)	35 (79.5)	94 (87.9)	83 (79.8)	44 (75.9)	14 (77.8)	20 (95.2)	24 (88.9)	0.23
Smoker (%)	8 (18.2)	12 (11.2)	6 (5.8)	14 (24.1)	1 (5.6)	5 (23.8)	3 (11.1)	0.015
Alcohol use (%)	28 (63.6)	52 (48.6)	70 (67.3)	29 (50.0)	8 (44.4)	6 (28.6)	21 (77.8)	0.001
White ethnicity (%)	42 (97.7)	97 (90.7)	91 (87.5)	49 (84.5)	16 (88.9)	18 (85.7)	26 (96.3)	0.70
Married (%)	25 (56.8)	49 (45.8)	52 (50.0)	21 (36.2)	12 (66.7)	8 (38.1)	16 (59.3)	0.48
University or postgraduate level of education (%)	19 (43.2)	39 (36.4)	50 (48.1)	14 (24.1)	7 (38.9)	4 (19.0)	17 (63.0)	0.014
IBS subtype (%)								
IBS‐C	1 (2.3)	29 (27.1)	20 (19.2)	5 (8.6)	11 (61.1)	2 (9.5)	24 (88.9)	
IBS‐D	27 (61.4)	21 (19.6)	31 (29.8)	32 (55.2)	1 (5.6)	7 (33.3)	1 (3.7)	
IBS‐M	16 (36.4)	53 (49.5)	50 (48.1)	20 (34.5)	5 (27.8)	12 (57.1)	2 (7.4)	
IBS‐U	0 (0)	4 (3.7)	3 (2.9)	1 (1.7)	1 (5.6)	0 (0)	0 (0)	< 0.001
IBS after acute enteric infection (%)	4 (9.1)	9 (8.4)	9 (8.7)	6 (10.3)	3 (16.7)	0 (0)	3 (11.1)	0.50
Most troublesome symptom (%)								
Abdominal pain	14 (31.8)	56 (52.3)	34 (32.7)	16 (27.6)	7 (38.9)	8 (38.1)	5 (18.5)	
Constipation	0 (0)	11 (10.3)	15 (14.4)	2 (3.4)	5 (27.8)	0 (0)	8 (29.6)	
Diarrhoea	15 (34.1)	7 (6.5)	14 (13.5)	11 (19.0)	0 (0)	5 (23.8)	0 (0)	
Bloating/distension	4 (9.1)	28 (26.2)	24 (23.1)	10 (17.2)	5 (27.8)	4 (19.0)	14 (51.9)	
Urgency	11 (25.0)	5 (4.7)	17 (16.3)	19 (32.8)	1 (5.6)	4 (19.0)	0 (0)	< 0.001
IBS‐SSS severity (%)								
Remission	0 (0)	0 (0)	12 (11.5)	1 (1.7)	0 (0)	0 (0)	0 (0)	
Mild	5 (11.4)	7 (6.5)	26 (25.0)	4 (6.9)	0 (0)	0 (0)	4 (14.8)	
Moderate	34 (77.3)	52 (48.6)	55 (52.9)	25 (43.1)	8 (44.4)	5 (23.8)	15 (55.6)	
Severe	5 (11.4)	48 (44.9)	11 (10.6)	28 (48.3)	10 (55.6)	16 (76.2)	8 (29.6)	< 0.001
Mean IBS‐SSS score (SD)	241.2 (63.0)	283.3 (68.0)	195.1 (80.2)	291.3 (84.3)	323.4 (62.2)	332.3 (52.9)	254.6 (72.9)	< 0.001
Met Rome IV criteria for IBS (%)	34 (77.3)	67 (62.6)	42 (40.4)	51 (87.9)	18 (100.0)	19 (90.5)	18 (66.7)	< 0.001
HADS‐anxiety categories (%)								
Normal	19 (43.2)	19 (71.8)	60 (57.7)	9 (15.5)	3 (16.7)	2 (9.5)	14 (51.9)	
Borderline abnormal	14 (31.8)	31 (29.0)	25 (24.0)	12 (20.7)	2 (11.1)	0 (0)	7 (25.9)	
Abnormal	11 (25.0)	57 (53.3)	19 (18.3)	37 (63.8)	13 (72.2)	19 (90.5)	6 (22.2)	< 0.001
Mean HADS‐anxiety score (SD)	8.1 (4.5)	11.1 (4.6)	7.1 (3.9)	12.3 (4.7)	12.6 (5.1)	13.5 (3.8)	7.9 (4.5)	< 0.001
HADS‐depression categories (%)								
Normal	38 (86.4)	52 (48.6)	87 (84.5)	17 (29.3)	6 (33.3)	6 (28.6)	19 (70.4)	
Borderline abnormal	4 (9.1)	27 (25.2)	14 (13.6)	22 (37.9)	3 (16.7)	5 (23.8)	5 (18.5)	
Abnormal	2 (4.5)	28 (26.2)	2 (1.9)	19 (32.8)	9 (50.0)	10 (47.6)	3 (11.1)	< 0.001
Mean HADS‐depression score (SD)	4.3 (3.0)	8.3 (4.2)	4.1 (3.1)	9.5 (4.2)	11.0 (5.4)	10.8 (4.4)	5.3 (4.1)	< 0.001
PHQ‐12 severity (%)								
Low	2 (4.5)	0 (0)	19 (18.3)	0 (0)	0 (0)	0 (0)	1 (3.7)	
Mild	23 (52.3)	4 (3.7)	55 (52.9)	4 (7.0)	0 (0)	0 (0)	12 (44.4)	
Moderate	18 (40.9)	65 (60.7)	30 (28.8)	38 (66.7)	4 (22.2)	0 (0)	12 (44.4)	
Severe	1 (2.3)	38 (35.5)	0 (0)	15 (26.3)	14 (77.8)	21 (100)	2 (7.4)	< 0.001
Mean PHQ‐12 score (SD)	7.2 (2.5)	12.1 (3.0)	5.8 (2.6)	11.1 (2.3)	15.6 (3.6)	17.2 (1.7)	8.0 (3.1)	< 0.001
Mean IBS‐QOL score (SD)	55.7 (21.0)	47.1 (21.7)	70.4 (17.5)	37.4 (19.5)	28.2 (16.8)	25.7 (13.8)	59.4 (20.7)	< 0.001

* *p* value for one‐way ANOVA for continuous data and Pearson *χ* ^2^ for comparison of categorical data.

### 3.2 Healthcare Use Among Individuals With IBS During 12‐Month Longitudinal Follow‐Up According to Cluster

Rates of discharge from clinic were generally lower among those in the four clusters with a high psychological burden, with the exception of cluster 5 (*p* = 0.05 for trend) (Table 3). There were no significant differences in rates of referral to a dietitian or a psychologist, but those in cluster 6 had the highest rates of referral for gut‐directed hypnotherapy (*p* = 0.01 for trend). Rates of prescribing a drug for IBS were highest in the four clusters with a high psychological burden, but also in cluster 7 (*p* = 0.001 for trend). The mean number of clinic appointments during 12‐month follow‐up was highest in those in clusters 5 and 6 (*p* = 0.037) and the mean number of drugs prescribed for IBS during 12‐month follow‐up was higher in clusters 2, 4, 5 and 6 (*p* < 0.001 for trend). In terms of drugs prescribed, as would be expected, patients in clusters 1 and 4 were the most likely to receive a drug for diarrhoea, but also cluster 6, and those in clusters 5 and 7 the most likely to receive a drug for constipation (*p* < 0.001 for trend for both). Use of antispasmodic drugs did not differ by cluster. Rates of use of gut‐brain neuromodulator use were lowest in clusters 6 and 7 (*p* = 0.037). The mean number of different drug types prescribed for IBS during 12‐month follow‐up was highest in the four clusters with the highest psychological burden (*p* < 0.001 for trend).

**TABLE 3:** Healthcare use among individuals with IBS during 12‐month longitudinal follow‐up according to cluster.

	Cluster 1: Diarrhoea and urgency with low psychological burden (*n* = 44)	Cluster 2: Low bowel symptom severity with abdominal pain and high psychological burden (*n* = 107)	Cluster 3: Low overall gastrointestinal symptom severity with low psychological burden (*n* = 104)	Cluster 4: Diarrhoea, abdominal pain and urgency with high psychological burden (*n* = 58)	Cluster 5: Constipation, abdominal pain and bloating with high psychological burden (*n* = 18)	Cluster 6: High overall gastrointestinal symptom severity with high psychological burden (*n* = 21)	Cluster 7: Constipation and bloating with low psychological burden (*n* = 27)	*p* *
Discharged from clinic (%)	17 (38.6)	32 (29.9)	47 (45.2)	16 (27.6)	8 (44.4)	3 (14.3)	11 (40.7)	0.05
Referred to a dietitian (%)	26 (59.1)	59 (55.1)	59 (56.7)	32 (55.2)	9 (50.0)	13 (61.9)	13 (48.1)	0.96
Referred to a gut‐directed hypnotherapist (%)	0 (0)	1 (0.9)	2 (1.9)	2 (3.4)	1 (5.6)	3 (14.3)	0 (0)	0.01
Referred to a psychologist (%)	19 (43.2)	49 (45.8)	36 (34.6)	29 (50.0)	11 (61.1)	10 (47.6)	11 (40.7)	0.32
Prescribed a drug for IBS (%)	29 (67.4)	87 (81.3)	66 (63.5)	52 (89.7)	17 (94.4)	16 (76.2)	22 (81.5)	0.001
Mean number of clinic appointments (SD)	1.7 (0.9)	1.6 (1.1)	1.5 (0.8)	1.8 (1.0)	2.2 (1.4)	2.0 (0.8)	1.5 (0.7)	0.037
Mean number of investigations (SD)	1.7 (1.3)	1.6 (1.6)	1.4 (1.4)	1.8 (1.6)	1.9 (1.8)	1.7 (1.7)	1.3 (1.5)	0.51
Mean number of drugs for IBS (SD)	1.3 (1.2)	1.8 (1.2)	1.2 (1.2)	1.8 (1.1)	2.4 (1.2)	1.5 (1.1)	1.3 (1.0)	< 0.001
Prescribed a drug for diarrhoea (%)	14 (31.8)	10 (9.3)	8 (7.7)	22 (37.9)	3 (16.7)	8 (38.1)	0 (0)	< 0.001
Prescribed a drug for constipation (%)	6 (13.6)	55 (51.4)	31 (29.8)	23 (39.7)	13 (72.2)	7 (33.3)	20 (74.1)	< 0.001
Prescribed an antispasmodic drug (%)	11 (25.0)	38 (35.5)	32 (30.8)	17 (29.3)	9 (50.0)	7 (33.3)	8 (29.6)	0.60
Prescribed a gut‐brain neuromodulator (%)	16 (36.4)	36 (33.6)	30 (28.8)	21 (36.2)	8 (44.4)	5 (23.8)	3 (11.1)	0.037
Mean number of different drug types for IBS (SD)^a^	1.1 (1.0)	1.3 (0.8)	1.0 (0.9)	1.4 (0.8)	1.8 (0.8)	1.3 (1.1)	1.2 (0.7)	0.001

^a^ Drug for diarrhoea or constipation, antispasmodic drug or gut‐brain neuromodulator.

* *p* value for one‐way ANOVA for continuous data and Pearson *χ* ^2^ for comparison of categorical data.

### 3.3 Healthcare Use Among Individuals With IBS During 12‐Month Longitudinal Follow‐Up According to Psychological Burden of the Cluster

Rates of discharge from clinic were significantly lower among those in a cluster with a high psychological burden (28.9% vs. 42.9%, *p* = 0.005) (Table 4) but there were no significant differences in rates of referral to a dietitian, psychologist or for gut‐directed hypnotherapy. Prescribing rates of a drug for IBS were also significantly higher among those in a cluster with a high psychological burden (84.3% vs. 67.2%, *p* < 0.001). Finally, the mean number of clinic appointments (*p* = 0.023), the mean number of drugs prescribed for IBS (*p* < 0.001), and the mean number of different drug types prescribed for IBS during 12‐month follow‐up were significantly higher among those in a cluster with a high psychological burden (*p* < 0.001 for trend).

**TABLE 4:** Healthcare use among individuals with IBS during 12‐month longitudinal follow‐up according to psychological burden of the cluster.

	Cluster with a high psychological burden^a^ (*n* = 204)	Cluster with a low psychological burden^b^ (*n* = 175)	*p* *
Discharged from clinic (%)	59 (28.9)	75 (42.9)	0.005
Referred to a dietitian (%)	113 (55.4)	98 (56.0)	0.91
Referred to a gut‐directed hypnotherapist (%)	7 (3.4)	2 (1.1)	0.15
Referred to a psychologist (%)	99 (48.5)	66 (37.7)	0.034
Prescribed a drug for IBS (%)	172 (84.3)	117 (67.2)	< 0.001
Mean number of clinic appointments (SD)	1.8 (1.1)	1.6 (0.8)	0.023
Mean number of investigations (SD)	1.7 (1.6)	1.4 (1.4)	0.11
Mean number of drugs for IBS (SD)	1.8 (1.2)	1.2 (1.1)	< 0.001
Mean number of different drug types for IBS (SD)	1.4 (0.9)	1.0 (0.9)	< 0.001

^a^ Clusters 2, 4, 5 and 6.

^b^ Clusters 1, 3 and 7.

* *p* value for independent samples *t*‐test for continuous data and Pearson *χ* ^2^ for comparison of categorical data.

## 4 Discussion

We recruited over 350 patients diagnosed with IBS in secondary and tertiary care, applied the LCA model that we derived previously, and assessed burden of IBS during 12 months of longitudinal follow‐up according to cluster membership at baseline. Despite having broadly similar demographics, patients in clusters with higher psychological burden (clusters 2, 4, 5 and 6), compared with those in clusters with lower psychological burden (clusters 1, 3 and 7), had more severe symptoms of IBS and lower IBS‐related quality of life at baseline. During follow‐up, rates of investigation were similar in both groups but those in clusters with higher psychological burden had higher rates of prescribed medications for IBS. There were no significant differences in rates of referral to a dietitian or a psychologist but those in cluster 6 (i.e., those with high overall gastrointestinal symptom severity with high psychological burden) had the highest referral rates for gut‐directed hypnotherapy. Finally, apart from those in cluster 5, rates of discharge from clinic were generally lower among those in the clusters with higher psychological burden. These observations held true, and strengthened, when patients were dichotomised into those in either a high or low psychological burden cluster at baseline.

This study recruited a large sample of patients with IBS from six hospitals in the UK. Although patients recruited in this study were seen by gastroenterologists with an interest in IBS, they were referred from primary care, implying that our results are likely to be generalisable to many patients with IBS in secondary or tertiary care. We obtained complete symptom data at baseline and data related to investigations, consultation rates, referral to other healthcare professions, medication use and discharge rates were extracted from patients' medical records over the 12‐month follow‐up period. All patients were diagnosed with IBS using a relatively standardised workup, as recommended by current UK guidelines [22]. Although gastroenterologists were able to ask questions about psychological symptoms and were able to access the completed patient questionnaire at baseline, cluster membership of patients was only assigned at the data analysis stage. Hence, it was not disclosed to either patients or gastroenterologists and could not have affected subsequent consultation behaviour, prescribing or management during follow‐up or data collection at 12 months. This means that it is unlikely that systematic bias due to cluster membership has been introduced.

Weaknesses of the study include the fact that the original LCA model applied to patients in secondary and tertiary care in the current study was derived from individuals in the community who met the Rome IV criteria for IBS [15]. However, we felt this was appropriate, as the characteristics of those with IBS diagnosed by a gastroenterologist were very similar to those diagnosed by a gastroenterologist and who also met Rome IV criteria. Overall, one‐third of patients who were felt to have IBS by a gastroenterologist did not meet the Rome IV criteria for IBS. This reflects their more stringent nature, which has increased their specificity for a diagnosis of IBS, and hence improved their performance as a diagnostic test [31]. In addition, the proportion of individuals in several of the seven clusters in the present study was very similar to that observed in the original cohort of individuals with Rome IV IBS in which the LCA was derived [15], and in a separate cohort of individuals with Rome IV IBS recruited from the community [16]. Although a range of treatments were examined, some therapies, such as gut‐directed hypnotherapy, were not available in all centres. Finally, despite recruiting a large sample, the number of patients in clusters 5, 6 and 7 was small, in keeping with the original LCA model, and this could have affected our results. This is supported by the fact that the associations we observed were strengthened when patients were dichotomised into those in either a high or low psychological burden cluster.

Two previous separate studies from our group have examined healthcare utilisation according to the clusters derived from this LCA model [16, 19]. One of these recruited 752 individuals with Rome IV‐defined IBS from the community in the UK [16], and the other applied the LCA model to 2195 individuals with Rome IV‐defined IBS in the Rome Foundation Global Epidemiological Study [19]. To the best of our knowledge, this study of healthcare utilisation according to LCA cluster in patients with IBS in secondary and tertiary care is, therefore, novel. Our finding that those in clusters with higher psychological burden had higher levels of medication use, compared with those in clusters with lower psychological burden, is similar to these previous studies [16, 19]. These previous studies also demonstrated that individuals with higher psychological burden had higher rates of IBS‐related consultation [16, 19]. In the present study, only those in two of the four clusters with higher psychological burden (clusters 5 and 6) had a higher mean number of clinic appointments. This finding could reflect the fact that we recruited a smaller number of patients, all of whom had at least one clinic appointment with a gastroenterologist. In contrast, the other studies were cross‐sectional and evaluated whether individuals with IBS, some of whom may have had a diagnosis for several years, had seen a gastroenterologist in the 12 months prior to study recruitment. In addition, in the current study these data were obtained directly from participants' medical records, whereas in the previous studies they were based on self‐report.

Our hypothesis that those in clusters with higher psychological burden, compared with those in clusters with lower psychological burden, would be more likely to be investigated was not supported by the results of our study. This remained the case even when we analysed data with all patients dichotomised into those in either a high or low psychological burden cluster at baseline. This is perhaps because, regardless of gastrointestinal or psychological symptoms reported by patients, gastroenterologists with an interest in IBS are making a positive diagnosis of IBS according to current UK guidelines [22], minimising investigations. Interestingly, there was no difference in rates of referral for psychological therapy among the seven clusters, and there may be several reasons for this. First, patients may not readily disclose psychological symptoms to gastroenterologists. Second, gastroenterologists may not routinely ask about psychological symptoms in a consultation that is, ostensibly, concerned with gastrointestinal health. Third, even when psychological symptoms are reported, gastrointestinal symptoms might be prioritised for treatment. Fourth, access to psychological or behavioural therapies may be limited or they may be used as a last resort when drug treatment has failed. Finally, patients may not be receptive to referral for psychological or behavioural approaches to treatment if this is not explained carefully, as they may feel it trivialises or dismisses the physical aspects of their illness. Conversely, dietary therapies are high on the patient agenda [32], and can be useful for managing global symptoms in IBS [33]. This might explain why dietitian referral rates did not differ between clusters, despite their contrasting symptom profiles.

The results of our study have important implications. Patients with IBS in clusters with a higher psychological burden generally had lower quality of life, higher levels of healthcare utilisation and lower discharge rates, compared with those with a lower psychological burden. Using these LCA clusters when first assessing patients with IBS in clinic would ensure that both gastrointestinal and psychological symptoms are assessed routinely, and at an early stage. This could help clinicians to identify and prioritise those most likely to benefit from specific treatment strategies. For example, whether gastrointestinal symptoms are predominant (clusters 1 and 7) or whether psychological symptoms (cluster 2) or both gastrointestinal and psychological symptoms (clusters 4, 5 and 6) need treatment. In the latter two instances, where psychological symptom burden is also high, peripherally acting drugs alone are unlikely to improve overall wellbeing and quality of life. Therefore, rather than waiting 12 months for these to fail before considering behavioural therapies, which is the current recommendation in the UK [22, 34], the use of these could be considered earlier, given their known efficacy in IBS [35, 36]. Similarly, patients with IBS in cluster 3, with both low gastrointestinal symptom severity and low psychological burden, may be able to be managed with lifestyle advice, reassurance and education about the disorder, with early discharge from secondary or tertiary care. However, there has been no study examining whether a more personalised treatment algorithm using LCA clusters can improve outcomes in the management of IBS. Future studies should seek to address this.

## Author Contributions

**Vivek C. Goodoory:** conceptualization, methodology, data curation, writing – original draft, writing – review and editing, formal analysis, project administration. **Mais Khasawneh:** conceptualization, methodology, data curation, writing – review and editing. **Christy Riggott:** data curation, writing – review and editing. **Dipesh H. Vasant:** conceptualization, data curation, methodology, writing – review and editing. **Maria Eugenicos:** conceptualization, data curation, methodology, writing – review and editing. **Imran Aziz:** conceptualization, writing – review and editing, data curation, methodology. **Maura Corsetti:** conceptualization, writing – review and editing, data curation, methodology. **Peter A. Paine:** conceptualization, data curation, methodology, writing – review and editing. **Ryan Danvers:** data curation, writing – review and editing. **Karen McKinnie:** data curation, writing – review and editing. **Debbie Bush:** data curation, writing – review and editing. **Christopher J. Black:** conceptualization, supervision, writing – review and editing, formal analysis, methodology. **Alexander C. Ford:** conceptualization, methodology, formal analysis, supervision, writing – review and editing, writing – original draft.

## Funding

The authors have nothing to report.

## Conflicts of Interest

Maura Corsetti: consulting fees from Opella (a Sanofi company), Biocodex, PROMEDCS, Nestle and Mayoly and research funding from Opella (a Sanofi company) and Aboca. Guarantor of the article: Alexander C. Ford. The other authors declare no conflicts of interest.

## Supporting information


**Data S1:** apt70583‐sup‐0001‐DataS1.docx.

## Data Availability

The data that support the findings of this study are available on request from the corresponding author. The data are not publicly available due to privacy or ethical restrictions.
